
JOURNAL INFORMATION
==============================
NLM Title Abbreviation: Behav Neurol
Journal ID: Behav Neurol
NLM Journal ID: 8914585
Journal ID: BN

Behavioural Neurology

ISSN: 0953-4180
EISSN: 1875-8584
Publisher: Wiley

ARTICLE INFORMATION
==============================
PMCID: PMC5434357
PMID: 21422561
DOI: 10.3233/BEN-2010-0301
Article ID: 312736
Article version: 1
Subjects: Research Article

Counterfactual Thinking and the Episodic System

Van Hoeck Nicole *
Ma Ning
Van Overwalle Frank
Vandekerckhove Marie
Vrije Universiteit Brussel Brussels Belgium
*Nicole Van Hoeck: nivhoeck@vub.ac.be
Print publication date: 2010
Electronic publication date: 2011 Mar 7
Volume: 23
Issue: 4
First page: 225
Last page: 227
Received 2011 Jan 31; Accepted 2011 Jan 31
Copyright: Copyright © 2010 Hindawi Publishing Corporation and the authors.
Copyright year: 2010
License: This is an open access article distributed under the Creative Commons Attribution License, which permits unrestricted use, distribution, and reproduction in any medium, provided the original work is properly cited.
License URL: https://creativecommons.org/licenses/by/3.0/

==============================
Behavioural Neurology 23 (2010) 225–227
DOI 10.3233/BEN-2010-0301
IOS Press

### Counterfactual thinking and the episodic system

Nicole Van Hoeck*, Ning Ma, Frank Van Overwalle and Marie Vandekerckhove
*Vrije Universiteit Brussel, Brussels, Belgium*

### 1. Introduction

When something bad happens, we often think how things could have turned out differently (e.g., "If I had set my alarm, I would not have missed my appointment."). Imagining an alternative reality, which could have replaced the actual one, is called *counterfactual thinking*. Counterfactual thoughts influence our feelings and thoughts about certain past events or actions. They also inform us how to prevent the past from repeating itself [5]. Therefore, counterfactual thinking is heavily intertwined with both past and future episodic thoughts.

Recent neurological studies show that episodic past and future thinking share several areas in the brain, which reflect common memory and social processes. A first set of areas is directly engaged by a variety of memory processes. Episodic memory (i.e., about specific events) activates the precuneus (PC) and nearby areas, including the retrosplenial cortex and the posterior cingulate cortex [4,9]. Semantic memory (i.e., general world knowledge) recruits the middle temporal gyrus (MTG) and also the temporal pole (TP), which plays, among other functions, an important role in representing social semantic concepts [6]. Relational processing in memory and the creation of new memory traces engages the (para-) hippocampus (HC [1]).

A second set of areas is involved in social cognition: thinking about the mind of oneself or other persons (*mentalizing*). According to Van Overwalle [10] mentalizing about temporary goals, intentions or desires of self or other persons activates the temporo-parietal junction (TPJ); while inferring more long-term social aspects (traits, norms, scripts) engages the medial prefrontal cortex (mPFC).

A last area, activated by the episodic thinking and related to both memory and social cognition, is the lateral prefrontal cortex (lPFC). Spreng et al. [7] suggest that this area is involved in keeping and manipulating information online.

Since this network of brain areas is not only activated during episodic memory tasks, but also during theory of mind tasks and rest periods (default mode), researchers propose that this brain network has a more general function of supporting the *projection* of oneself in another temporal or contextual situation (including in another person), or of scene construction [7]. Since counterfactual thinking implies self-projection and scene construction, it should also activate this brain network (Hypothesis 1)!

To investigate the common and different (sub-)areas involved in counterfactual thinking and episodic memory, we directly compared brain activity during episodic thinking (past retrieval and future construction) and counterfactual reasoning using event-related functional MRI. Since there are different types of counterfactuals, we focused on the most frequent one in daily life [5] in which an event turns out better (upward) by replacing or adding a different action. Based in part on the predictions by Barbey et al. [3], we expect that these counterfactuals, in contrast to thoughts about a negative past, will generate greater activation in a number of areas (Hypothesis 2). Undoing the past by one's own or other person's effort involves a goal-directed action which will activate the TPJ to a greater extent. The anticipation of positive outcomes and the emotional processing of feelings of regret will activate the orbitofrontal cortex (OFC; is involved in emotion regulation by representing the anticipated event outcomes).

*Corresponding author. E-mail: nivhoeck@vub.ac.be.

ISSN 0953-4180/10/$27.50 © 2010 – IOS Press and the authors. All rights reserved

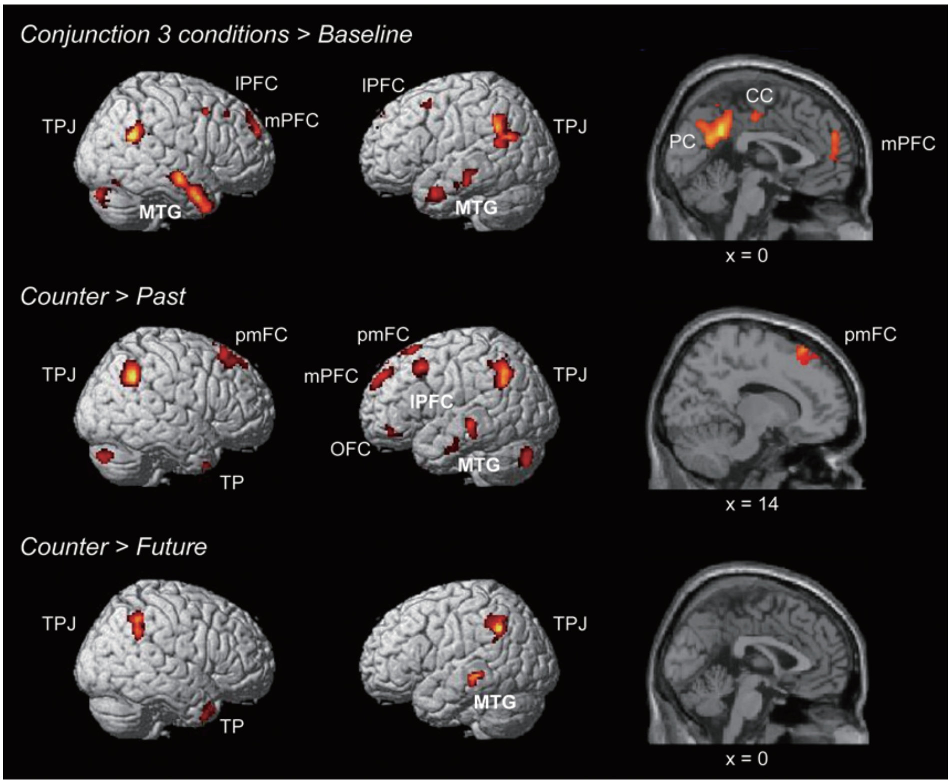

Fig. 1. Whole brain analysis with threshold at $p < 0.001$ and $> 31$ voxels (see text).

The conflict between the counterfactual and past reality will engage areas involved in conflict monitoring and control, such as the posterior medial Frontal Cortex (pmFC) and lateral PFC (lPFC [2]). In addition, we expect little differences in activation pattern between counterfactuals and positive future thoughts. Both are in this study positive in their nature, in conflict with the past, and ask for behavioral planning. Nevertheless, counterfactuals specifically focus on undoing the event by someone's actions. Therefore, we expect them to activate the TPJ more (Hypothesis 3).

### 2. Method

One week before scanning, 14 healthy participants (six males and eight females, mean age 21 years) were asked to describe negative events from their own past. For each participant 20 of their stories were adapted into personalized trials which were used during scanning. Three relevant cue words were added to each story to be used as a memory aid (e.g., car – phone – holiday).

During scanning participants had to recall a negative past event, imagine a similar positive future event, or imagine a similar counterfactual event (better outcome than the past reality). In a semantic baseline condition, they had to recall as much detail as possible about the semantic meaning of each cue word. Each condition consisted out of 20 trials. Each trial began with a slide announcing the condition (task), the three personal cue words and specific task instruction (e.g. "Imagine what would have happened if you had paid more attention to the road during the car trip in France").

We report statistical contrasts after correction for multiple comparisons, using the non-parametric test statistic developed by Slotnick et al. [8]. After 2000 simulations a cluster extent was determined at 31 contiguous resampled voxels which is equivalent to a corrected $p = 0.05$.

Counterfactual thinking and episodic retrieval activated common (TPJ, HC, MTG, PC, lPFC, mPFC) as well as distinct brain areas (TP, OFC, pmFC). All three hypothesis were confirmed (see Fig. 1), although the (para)HC only survived a cluster threshold of 10 voxels. Nevertheless, we did not predict that counterfactual thinking would activate the MTG and TP more than episodic thinking. A possible explanation is that in order to imagine a better outcome for negative past event, participants also retrieved information about what behavior is more appropriate according to social conventions and semantic world knowledge, resulting in the greater activation of the MTG and TP.

We can conclude that counterfactual thinking engages a similar brain network as episodic memory. Nonetheless, it also activates different brain areas, especially in contrast to past episodic retrieval, since counterfactual thinking requires participants to monitor different (and conflicting) realities (pmFC & lPFC), anticipate an upward improvement (OFC) and have the intention to bring about that change (TPJ).

### References

[1] D.R. Addis, A.T. Wong and D.L. Schacter, Remembering the past and imagining the future: common and distinct neural substrates during event construction and elaboration, *Neuropsychologia* **45** (2007), 1363–1377.

[2] M.M. Botvinick, J.D. Cohen and C.S. Carter, Conflict monitoring and anterior cingulate cortex: An update, *Trends in Cognitive Sciences* **8** (2004), 539–546.

[3] A.K. Barbey, F. Krueger and J. Grafman, Structured event complexes in the medial prefrontal cortex support counterfactual representations for future planning, *Philosophical Transactions of the Royal Society B* **364** (2009), 1291–1300.

[4] R.L. Buckner and D.C. Carroll, Self-projection and the brain, *TRENDS in Cognitive Sciences* **11**(2) (2006), 49–57.

[5] K. Epstude and N.J. Roese, The Functional Theory of Counterfactual Thinking, *Personality and Social Psychology Review* **12** (2008), 168–192.

[6] L.A. Ross and I.R. Olson, Social cognition and the anterior temporal lobes, *Neuroimage* **49** (2010), 3452–3462.

[7] R.N. Spreng, R.A. Mar and A.S.N. Kim, The Common Neural Basis of Autobiographical Memory, Prospection, Navigation, Theory of Mind, and the Default Mode: A Qauntitave Meta-analysis, *Journal of Cognitive Neuroscience* **21**(3) (2008), 489–510.

[8] S.D. Slotnick, L.R. Moo, J.B. Segal and J. Hart, Distinct prefrontal cortex activity associated with item memory and source memory for visual shapes, *Cognitive Brain Research* **17** (2003), 75–82.

[9] M.M. Vandekerckhove, H.J. Markowisch, M. Mertens and F.G. Woermann, Bi-hemispheric engagement in the retrieval of autobiographic episodes Bi-hemispheric engagement in the retrieval of autobiographical episodes, *Behavioral Neurology* **16**(4) (2005), 203–210.

[10] F. Van Overwalle, Social cognition and the brain: A meta-analysis, *Human Brain Mapping* **30** (2009), 829–858.
